# Inside the Interaction: Contact With Familiar Humans Modulates Heart Rate Variability in Horses

**DOI:** 10.3389/fvets.2020.582759

**Published:** 2020-11-30

**Authors:** Chiara Scopa, Alberto Greco, Laura Contalbrigo, Elisabetta Fratini, Antonio Lanatà, Enzo Pasquale Scilingo, Paolo Baragli

**Affiliations:** ^1^Italian National Reference Centre for Animal Assisted Interventions, Istituto Zooprofilattico Sperimentale delle Venezie, Legnaro, Italy; ^2^Department of Information Engineering, University of Pisa, Pisa, Italy; ^3^Feel-Ing s.r.l., Pisa, Italy; ^4^Department of Information Engineering, University of Florence, Florence, Italy; ^5^Department of Veterinary Sciences, University of Pisa, Pisa, Italy; ^6^Research Center “E. Piaggio,” University of Pisa, Pisa, Italy

**Keywords:** *Equus caballus*, human–animal relationship, inter-specific interaction, heartbeat dynamics, autonomic nervous system, emotional valence

## Abstract

A human–animal relationship can be developed through subsequent interactions, affected by the positive or negative emotional valence of the proceeding one. Horses implement a process of categorization to classify humans with whom they interact as positive, negative, or neutral stimuli by evaluating the kind of approach and the nature of the contact. In these terms, human–animal interactions are emotionally charged events, eliciting specific emotional states in both subjects involved. Although the human–horse relationship has been mainly investigated through behavioral analysis, physiological indicators are needed for a more objective assessment of the emotional responses. Heart rate variability (HRV) is a commonly used autonomic nervous system (ANS) correlate estimating the sympathovagal balance as a psychophysiological marker of emotion regulation in horses. We have assumed that long-term positive relationships with humans may have a positive and immediate impact on the emotional arousal of the horse, detectable, *via* ANS activity, during the interaction. We analyzed horses' heartbeat dynamics during their interaction with either familiar or unfamiliar handlers, applying a standardized experimental protocol consisting of three different conditions shifting from the absence of interaction to physical contact. The ANS signals were monitored through an innovative non-invasive wearable system, not interfering with the unconscious emotional response of the animal. We demonstrated that horses appeared to feel more relaxed while physically interacting (e.g., grooming on the right side) with some familiar handlers compared to the same task performed by someone unfamiliar. The shift of the sympathovagal balance toward a vagal predominance suggests that the horses experienced a decrease in stress response as a function not only of the handler's familiarity but also of the type of interaction they are experiencing. These results constitute the objective evidence of horses' capacity to individually recognize a familiar person, adding the crucial role of familiarity with the handler as a paramount component of human–animal interaction. Our rigorous methodological approach may provide a significant contribution to various fields such as animal welfare while further investigating the emotional side of the human–animal relationships.

## Introduction

Horses can discriminate between familiar and unfamiliar humans using both visual and vocal cues (1, 2); they are also able to form a long-lasting memory of a specific subject (3, 4). This ability suggests that the level of familiarity can affect horses' tendency to engage again with the same human (5), also allowing these animals to recognize their caretakers long after the last encounter (6).

Indeed, human companions have a greater chance of leaving a positive image in horses' memory if their behavior is appropriate starting from the first approach. This may occur during training procedures or stable management (7), which the animal may recall for several months (8). The human–animal relationship is built on a succession of basic interactions, and the “positive” or “negative” valence of each interaction determines the occurrence of the next one (9). Therefore, by evaluating attitudes, kinds of approach, temperament, and the nature of the last contact, horses are able to implement a “categorization” process in order to label humans as positive, negative, or neutral stimuli (10). The motivation to react to perceived stimuli has an adaptive value, eliciting approaching behaviors toward survival sources or triggering avoidance in those situations perceived as a threat. These motivational factors, affecting the probability to move toward or away from stimuli (approach/avoidance), are significantly correlated with the valence (i.e., pleasantness) and the arousal (i.e., perception intensity) of the stimulus, as the two main components of emotion perception (11). Any emotional event can be either positive or negative. These tags, embodying emotive valence, differ in how they arouse an individual (11, 12). Similarly, human–animal interactions can be considered emotionally charged events, the positive/negative valence of which determines the ultimate quality (13). Investigating the emotional side of the human–animal bond can provide stimulating insights into animal cognition and social behavior. Hence, emotions affect communication with others, which constitutes a building block of the evolution of social species. This approach has generated detailed studies on behavioral and physiological indicators of emotions [e.g., (14–16)]. In prey species such as horses, visible behavioral markers of fear or distress may run counter to their survival strategy (17, 18). Although behaviors provide an immediate way to determine the response of an animal to environmental factors, the accurate interpretation of behavioral signals needs to be corroborated by physiological indicators (19, 20). Emotions, in fact, are expressed through a set of coordinated responses, including physiological signals (21, 22), which are affected by the social interaction and may determine its outcomes. In the case of horses, for example, the nature of their interaction with humans, which may shift from occasional management to a more intimate bond in daily contact, is reflected in their physiological and emotional responses. The most used autonomic nervous system (ANS) correlations for behavioral assessment are heart rate and heart rate variability (HRV). Heart rate corresponds to the number of heart beats per unit of time, and these beats are slowed down or accelerated by parasympathetic activity or sympathetic activity, respectively. HRV describes normal fluctuations in the time intervals of consecutive heartbeats, thus reflecting the interplay between the sympathetic and parasympathetic nervous systems. In particular, HRV indicates the shift from an autonomic balance toward a sympathetic dominance, adding extra information about individual temperament and reactivity to stimuli (23). Changes in the ANS have been increasingly used as an indicator of stress level in many species as a way to further employ this approach to animal behavioral assessment. With regard to companion animals, such as horses, the sympathovagal balance as a psychophysiological marker of emotion can be estimated *via* HRV (24, 25). Scientific evidence indicates that a modification in the time interval between successive heartbeats may imply a neurophysiological response to stress (24–29). The current challenge is to find a way to define the human–horse relationship by measuring its multifaceted aspects, particularly on the level of familiarity connecting the participants and the emotional valence punctuating the whole experience. In the present study, we hypothesize that long-term positive relationships with humans may have a positive and immediate impact on the emotional arousal of the horse. We expect the ANS activity of the horse to reflect a relaxed psychophysiological state while it experiences a familiar human interaction. To verify this hypothesis, we analyzed the heartbeat dynamics of horses during their interactions with both familiar and unfamiliar handlers. To this aim, we selected familiar people from among those who are mainly involved in the horse's daily activities such as management or training. To represent unfamiliar humans, we recruited people who were already familiar with horses but were unknown to our test subjects. Standardized interaction tests between humans and horses were designed to understand how horses perceive physical closeness and being handled by a human.

## Materials and Methods

### Ethical Statement

The study was performed in accordance with the ethical standards of the Declaration of Helsinki and with the recommendations of the Italian Animal Care Act (Decree Law 26/2014). The Ethical Committee on Animal Experimentation of the Experimental Zooprophylactic Institute of Venice (IZSVe) approved the experimental protocol in each of its parts (i.e., handling procedures, data collection methods, CE IZSVe 07/2020). Human subjects were enrolled on a voluntary basis, and they signed an informed consent statement to take part in the study. They were advised about their rights, data management, and protection in accordance with the Reg. EU N. 679/2016. The horses' owners gave written consent to the use of their horses in this experiment.

### Animal Subjects

We selected 23 mixed-breed horses (mean 14 ± 6.98 SE years old, nine mares and 14 geldings) from three different stables, all located in Italy (see Table 1 for details). All enrolled horses were in good health and showed no signs of injury. Exclusion criteria included the presence of any abnormal behaviors or stereotypes or the horse's involvement in any kind of professional equestrian sports. We chose participating stables based on management standards, including handling procedures and riding activities. In particular, we evaluated the primary activities undertaken by each horse, their daily workload, the number of people they were used to interacting with during activities and/or for management, their social life with conspecifics, and feeding management. Selected subjects were mostly involved in amateur-level riding activities with up to 3 h of ridden or ground work per day. The horses were accustomed to interactions with two to six people for daily management and to many more for the aforementioned activities. We accepted horses group-housed in paddocks, provided they spent short periods of time in a single stall as needed. This allowed us to exclude the possibility of inducing stress during the experimental tasks that could arise from being isolated in a box away from the social group. All subjects had free access to water. Pastures were supplemented with hay; some horses received concentrated feed and/or small amounts of vegetables.

**Table 1:** Horses selected from three different stables.

**Individual**	**Stable**	**Sex**	**Breed**	**Age**
Ckendy	NPP	F	Haflinger	20
Dado	NPP	G	Sardinian anglo-arabian	12
Didol	NPP	G	Argentino	23
Friso	NPP	G	Friesian	8
Ivan	NPP	G	Belgian double pony	27
Neve	NPP	F	Camargue	21
Remy	NPP	G	Haflinger	14
Arabella	AE	F	Sella italiano	28
Arramon	AE	G	Haflinger	19
Betta	AE	F	Arabian	9
Danilù	AE	G	Sella italiano	10
Dragonhair	AE	G	Sella italiano	10
Ercole	AE	G	Friesian	13
Falco	AE	G	Maremmano	13
Oliver	AE	G	Haflinger	11
Saif	AE	G	Arabian	8
Sunny	AE	F	Hanoverian	21
Erika	RdC	F	Monterufoli	8
Ilex	RdC	G	Monterufoli	4
Gelso	RdC	G	Monterufoli	6
Ginepra	RdC	F	Monterufoli	6
Uga	RdC	F	Monterufoli	15
Ginestra	RdC	F	Monterufoli	6

*NPP, Nero Per Passione; AE, Addestramento Etologico; RdC, Riserva di Cornocchia. F, female; G, gelding*.

### Human Subjects

We recruited human volunteers from different equestrian establishments between May and September 2019, on a network basis of personal contacts who themselves recruited volunteers in their respective locations and from their horse-owning contacts. We enrolled 22 subjects overall (mean 35.36 ± 13.17 SE years old; 12 females, 10 males). Among them, 12 people participated in the study as familiar persons and 10 as unfamiliar ones. To each familiar person, an unfamiliar same-sex person was matched. None of the involved human participants had any background of psychiatric or psychological disorders. All handlers were required to have experience with and be confident in handling horses. The unfamiliar handlers were a convenience sample of people who were present at the location or, at the time of the study, were not familiar with the horse to be tested. All humans involved in the trials wore similar clothes (specifically, blue jeans and a blue long-sleeved shirt) during the tasks. Starting 1 week before the start of the experiment, they were all required to use the same odorless neutral pH products. This procedure helps exclude the bias of familiar body odors' recognition.

### Protocol of Interaction

The experimental protocol, modified after (30, 31), consisted of an interaction task with three different conditions, each one lasting 5 min, combining a familiar/unfamiliar human handler test with the concomitant recording of horses' ECGs. The order of interactions with familiar/unfamiliar humans was randomized.

**Session 1 (S1)** – During the first phase, the human subject and the horse were left alone in separate areas. The horse was left free to move in its own familiar stall (4 ×4 m) (32), while the person was standing in the stable's service room. This session was considered the resting phase to collect basal ECG signals.

**Session 2 (S2)** – Successively, human subjects moved from the service room to the stall of the horse itself. They entered, without other humans, and placed themselves near the door and stood still, staring at the floor. In the meantime, the horse was still free to move and explore the environment. This phase implied both visual and olfactory interactions. In this session, the horse controlled interactions, deciding whether to approach, sniff, touch, or stay away from the human.

**Session 3 (S3)** – At the end of session 2, the human subject took a brush previously positioned outside the box, within arm's reach. He or she approached the horse to brush it. The grooming session lasted 2.5 min on each side (S3L left side and S3R right side) in a randomized order among the subjects. If the horse tried to move, the person had to maintain contact with it to keep on with the grooming procedure. Unlike the previous phase, this time the person had control over the interaction, constantly seeking connection with the animal. The horse could not avoid the interaction.

### ECG Signal Collection

The ANS response plays an essential role in the study of the familiar vs. unfamiliar horse interplay; therefore, in the experimental phase, the ECGs of both humans and horses were monitored through two wearable systems (33, 34).

**Figure 1:**
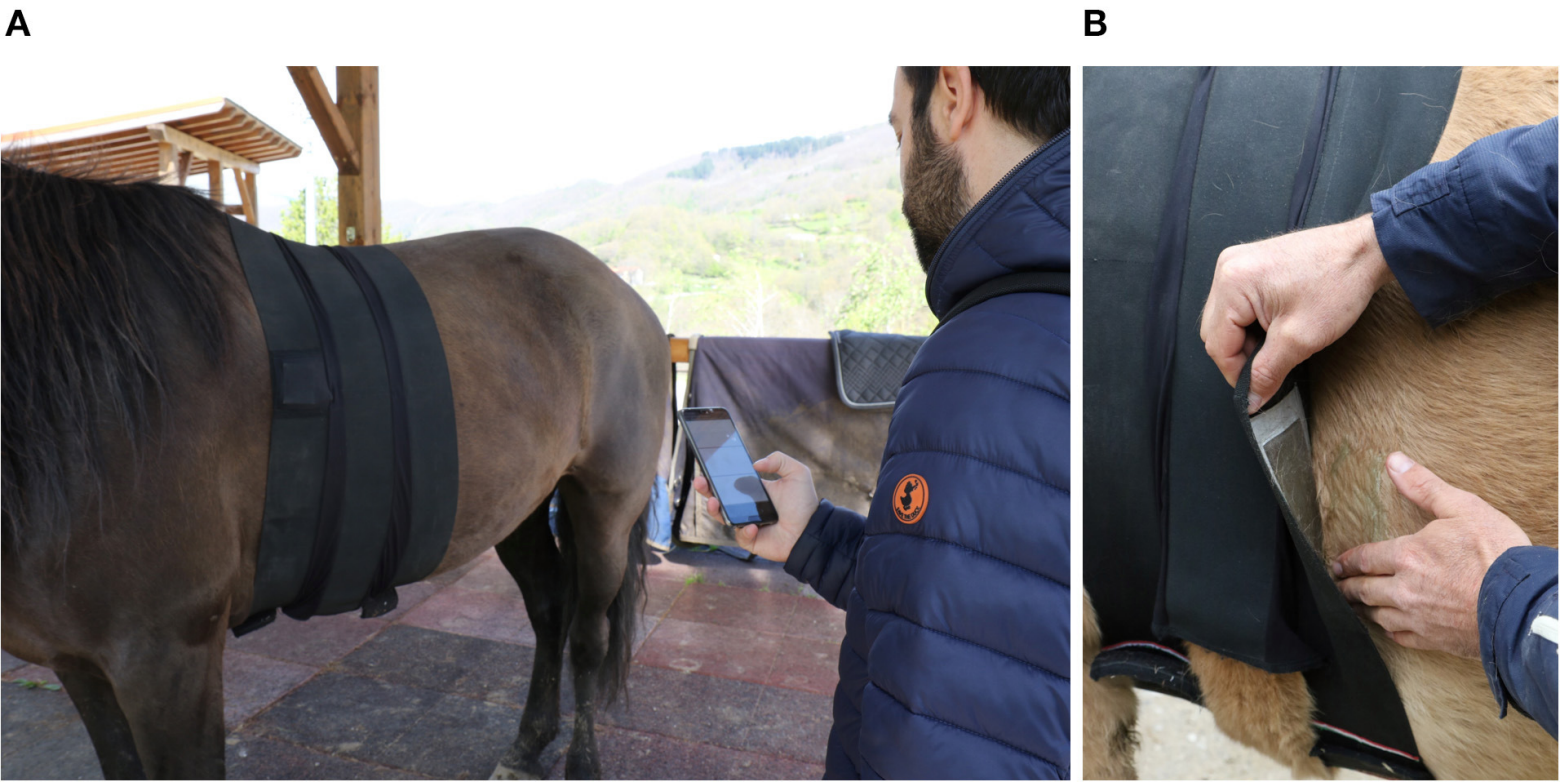
The monitoring system worn by tested horses. In **(A)**, the belt placed on the horse's chest and wirelessly controlled by a mobile app; in **(B)**, one of the electrodes integrated in the elastic belt. Photo by S. Seganfreddo.

Comfort and strong adaptability to experimental conditions are just a few of the advantages the wearable systems showed. Moreover, the systems developed by the University of Pisa for both humans and animals guaranteed a suitable solution for ANS monitoring without interfering with the hidden and unconscious emotional responses arising from the human–horse interaction. The belt used for horses was specifically designed to not be more intrusive than a saddle or any similar riding equipment, and the functionality of the belt has been previously validated (35, 36). Particularly, the two textile-based monitoring systems (37) recorded ECGs on a sampling frequency equal to 250 Hz. The two systems present a similar configuration with two electrodes composed of conductive yarn and one textile stretchable respiration sensor, completely integrated in a textile belt surrounding the body of either human or horse (Figure 1). In addition, a Bluetooth Low Energy (BLE) connection and a long-life battery supply allowed continuous monitoring of the physiological signals. Before starting the S1, both the human and the animal subjects were habituated to the systems for ~5 min. During this time window, the functionality of the remote control app was also tested.

### Data Analysis and Statistics

#### Heart Rate Variability Analysis

For the ECG and HRV analysis, we employed Kubios HRV analysis software (Biosignal Analysis and Medical Imaging Group at the Department of Physics, University of Kuopio, Kuopio, Finland) and MATLAB R2019 (The MathWorks, Inc.). The first step in the processing of the ECG signals is to determine the R-peaks of the QRS complexes. To this aim, we applied the Kubios built-in QRS detector algorithm based on the Pan–Tompkins method. Accordingly, each ECG was preprocessed through a bandpass filter in the frequency band of 0.05–40 Hz to reduce power line noise, baseline wander, and other noise components, a squaring of the data samples (to highlight R peaks and help the QRS detection) and a moving average filter (with a window width of 150 ms to smooth close-by peaks). The decision rules included amplitude threshold and comparison to expected values between adjacent R-waves. The threshold was adapted every time a new R-peak was detected. Furthermore, possible ectopic or misdetected R-peaks in the derived R-R time series were corrected after visual inspection of each tachogram. Due to the high quality and signal-to-noise ratio of the recorded ECG data, only <0.15% of the R-peaks on average were manually added or removed from the estimated tachogram. Accordingly, the resulting R-R time series did not require any algorithmic preprocessing step, and no outlier signal segments (i.e., excessive artifacts) were excluded for further analyses, but only small manually corrections were applied. The estimated series of R-R intervals were used to calculate the indexes of HRV in both time and frequency domains (38). The extracted HRV metrics aimed at quantifying the cardiovascular dynamics over time to infer with the horse psychophysiological state. Specifically, within each experimental session, we computed the mean value (μRR) and the standard deviation (σRR) of the RR interval series. Given the analogy between σRR and the total power spectrum, it reflects all the cyclic components responsible for variability in the time window. In addition, as recommended by the Task Force of the European Society of Cardiology and the North American Society of Pacing and Electrophysiology (39), we computed further standard HRV metrics such as the square root of the mean squared differences between successive RR intervals (RMSSD) and the percentage of consecutive R-R interval differences >50 ms (pNN50). In the frequency domain, we used an autoregressive modeling-based method to estimate the HRV spectra (AR spectrum). The order of the AR model was set up to the default value of 16 (40). Of note, before computing the AR spectrum, the non-evenly sampled R-R interval series were firstly interpolated by means of a cubic spline function. From each AR spectrum, according to the literature (24, 41, 42), we defined two main HRV spectral bands: the low-frequency band (LF, from 0.01 to 0.07 Hz) and the high-frequency band (HF, from 0.07 to 0.6 Hz). The frequency bandwidths were adapted from studies on human heartbeat dynamics to the horse spectral dynamics in order to reflect the sympathovagal nerve activity. Particularly, the HF components of the HRV band are assumed to be solely influenced by the parasympathetic nervous system. In contrast, the LF band is influenced by both the sympathetic and parasympathetic nervous systems. Once LF and HF ranges were defined, we computed the power spectrum in both LF and HF bands (LF power and HF power), the LF and HF frequency peaks (LFpeak and HFpeak), the LF and HF power normalized to the sum of LF + HF power (LFnu and HFnu), the power in LF band and HF band expressed as a percentage of the total power (LFpower % and HFpower %), and the ratio between LF and HF power (LF/HF). It is worthwhile noting that the LF/HF ratio, which has been frequently used in the scientific literature to assess the sympathetic and parasympathetic balance, has not been fully accepted as an accurate measure of the ANS balance since the LF band also contains parasympathetic dynamics.

#### Statistical Analyses

According to the experimental paradigm described in the Protocol of Interaction section, each horse performed the same tasks in two different experimental conditions: one while interacting with the familiar human handler and the other one while interacting with an unfamiliar one. Accordingly, each feature was calculated for each experimental session (i.e., S1, S2, S3R, and S3L) in both conditions. Afterward, normalization based on the S1 values, considered baseline, was applied to each feature computed within the S2, S3R, and S3L sessions in order to study the perturbation induced by both the visual and the olfactory interaction (S2n) and also by the human brush (S3Rn and S3Ln) on horse heartbeat dynamics. On the normalized features, two statistical analyses were performed: an intra-set analysis (Φ1) (both for the familiar and the unfamiliar interaction groups) and an inter-group analysis (Φ2). The Shapiro–Wilk test rejected the null hypothesis of Gaussian distribution of the feature samples; therefore, non-parametric statistical tests were adopted.

Φ 1) First, we applied a Friedman test to investigate statistical differences among the three experimental sessions (S2n, S3Rn, and S3Ln) within both familiar and unfamiliar interaction groups. In *post-hoc* analysis, each pair of sessions was compared with each other using a Bonferroni-corrected Wilcoxon signed-rank test to determine significant differences of each pairwise comparison.
Φ 2) Secondly, we investigated statistical differences between the “familiar interaction” and “unfamiliar interaction” for each normalized experimental session S2n, S3Ln, and S3Rn using a Bonferroni-corrected Wilcoxon signed-rank test.

Of note, Friedman test p-values were adjusted through a false discovery rate (FDR) procedure for multiple hypotheses testing (43). Together with the p-value, we reported the effect size of each Wilcoxon signed-rank test (*r* = $Z/\sqrt{N}$, where *Z* represents the value of the z-statistics and *N* is the total number of observations).

## Results

In Table 2, the median and median absolute deviation (MAD) are reported, calculated among all the horses for each HRV metrics of every experimental task (S2, S3L, S3R normalized by S1) in both experimental conditions (familiar and unfamiliar interactions). Moreover, the *p*-values in Table 2 represent both the intra- and inter-group statistical results.

**Table 2:** Median ± median absolute deviation (MAD) of all normalized features computed in each session and during the interaction with both the familiar and unfamiliar humans.

**Feature**	**Session**	**Median ± MAD** **Horse–familiar** **person**	**Median ± MAD** **Horse–unfamiliar** **person**	**Φ2** ***P*-values**
μRR	S2n	1.03 ± 3.83e-02	1.04 ± 3.79e-02	*p* = 0.976
	S3Ln	1.06 ± 4.99e-02	1.08 ± 6.49e-02	*p* = 0.484
	S3Rn	1.05 ± 5.35e-02	1.05 ± 5.40e-02	*p* = 0.162
	**Φ1 Friedman** ***p*****-value**	***p*** **= 1.41e-03**	***p*** **= 7.68e-03**	
σRR	S2n	1.07 ± 0.37	1.32 ± 0.47	*p* = 0.976
	S3Ln	0.81 ± 0.20	0.75 ± 0.24	*p* = 0.429
	S3Rn	0.87 ± 0.22	0.79 ± 0.29	*p* = 0.831
	**Φ1 Friedman** ***p*****-value**	***p*** **= 8.36e-04**	***p*** **= 4.56e-03**	
RMSSD	S2n	0.97 ± 7.04e-02	0.92 ± 0.14	*p* = 0.927
	S3Ln	0.94 ± 8.68e-02	0.87 ± 0.17	*p* = 0.605
	S3Rn	1.03 ± 0.21	0.91 ± 0.17	*p* = 0.362
	Φ1 Friedman *p*-value	*p* = 0.840	*p* = 8.39e-02	
pNN50	S2n	0.95 ± 0.14	0.95 ± 0.11	*p* = 0.738
	S3Ln	1.03 ± 0.21	0.92 ± 0.11	*p* = 0.584
	S3Rn	0.99 ± 0.21	0.93 ± 0.19	*p* = 0.181
	Φ1 Friedman *p*-value	*p* = 0.663	*p* = 0.438	
LFpeaκ	S2n	0.80 ± 0.20	1.00 ± 0.33	*p* = 0.101
	S3Ln	0.86 ± 0.26	1.00 ± 0.33	*p* = 0.897
	S3Rn	1.00 ± 0.40	1.00 ± 0.50	*p* = 0.263
	Φ1 Friedman *p*-value	*p* = 0.762	*p* = 0.753	
LFpower	S2n	0.84 ± 0.40	1.28 ± 0.66	*p* = 0.808
	S3Ln	0.87 ± 0.55	0.48 ± 0.36	*p* = 0.212
	S3Rn	0.93 ± 0.67	0.99 ± 0.68	*p* = 0.761
	**Φ1 Friedman** ***p*****-value**	*p* = 0.309	***p*** **= 0.0327**	
LFpower%	S2n	1.01 ± 0.30	0.95 ± 0.24	*p* = 0.693
	S3Ln	1.14 ± 0.35	0.92 ± 0.30	*p* = 0.236
	S3Rn	0.98 ± 0.24	1.05 ± 0.24	*p* = 0.543
	Φ1 Friedman *p*-value	*p* = 0.499	*p* = 0.260	
LFnu	S2n	0.99 ± 0.14	1.03 ± 0.17	*p* = 0.879
	S3Ln	1.04 ± 0.20	0.93 ± 0.14	*p* = 0.301
	S3Rn	0.94 ± 0.11	1.05 ± 0.18	*p* = 0.162
	Φ1 Friedman *p*-value	*p* = 0.296	*p* = 0.0646	
HFpeak	S2n	1.00 ± 0.20	1.02 ± 0.19	*p* = 0.316
	S3Ln	1.00 ± 0.28	1.10 ± 0.25	*p* = 0.927
	S3Rn	1.07 ± 0.24	1.04 ± 0.41	*p* = 0.592
	Φ1 Friedman *p*-value	*p* = 0.703	*p* = 0.904	
HFpower	S2n	0.84 ± 0.34	0.81 ± 0.20	*p* = 0.212
	S3Ln	0.82 ± 0.22	0.48 ± 0.26	*p* = 0.094
	S3Rn	1.11 ± 0.28	0.55 ± 0.30	***p*** **= 3.50e-03**
	Φ1 Friedman *p*-value	*p* = 0.296	*p* = 0.0705	
HFpower%	S2n	0.88 ± 0.58	0.64 ± 0.42	*p* = 0.648
	S3Ln	1.29 ± 0.75	1.13 ± 0.31	*p* = 0.563
	S3Rn	1.22 ± 0.54	0.77 ± 0.36	***p*** **= 0.044**
	**Φ1 Friedman** ***p*****-value**	***p*** **= 7.68e-03**	***p*** **= 0.0260**	
HFnu	S2n	1.16 ± 0.48	0.92 ± 0.44	*p* = 0.693
	S3Ln	0.84 ± 0.40	1.13 ± 0.45	*p* = 0.879
	S3Rn	1.22 ± 0.46	0.69 ± 0.31	*p* = 0.059
	Φ1 Friedman *p*-value	*p* = 0.296	*p* = 0.0646	
LF/HF	S2n	0.79 ± 0.52	1.12 ± 0.71	*p* = 0.952
	S3Ln	1.40 ± 0.87	0.81 ± 0.35	*p* = 0.670
	S3Rn	0.76 ± 0.38	1.60 ± 1.08	*p* = 0.181
	Φ1 Friedman *p*-value	*p* = 0.296	*p* = 0.0646	

P-values in the last column show the results of the Φ2 statistical analysis. Results of the Φ1 comparisons among sessions are shown in the rows denoted as “Φ1 Friedman p-values” for both familiar and unfamiliar groups.

μRR, mean value of the RR interval series; σRR, standard deviation of the RR interval series; HF, high-frequency band; LF, low-frequency band; RMSSD, square root of the mean squared differences between successive RR intervals.

*Bold values represent statistically significant p-values*.

**Figure 2:**
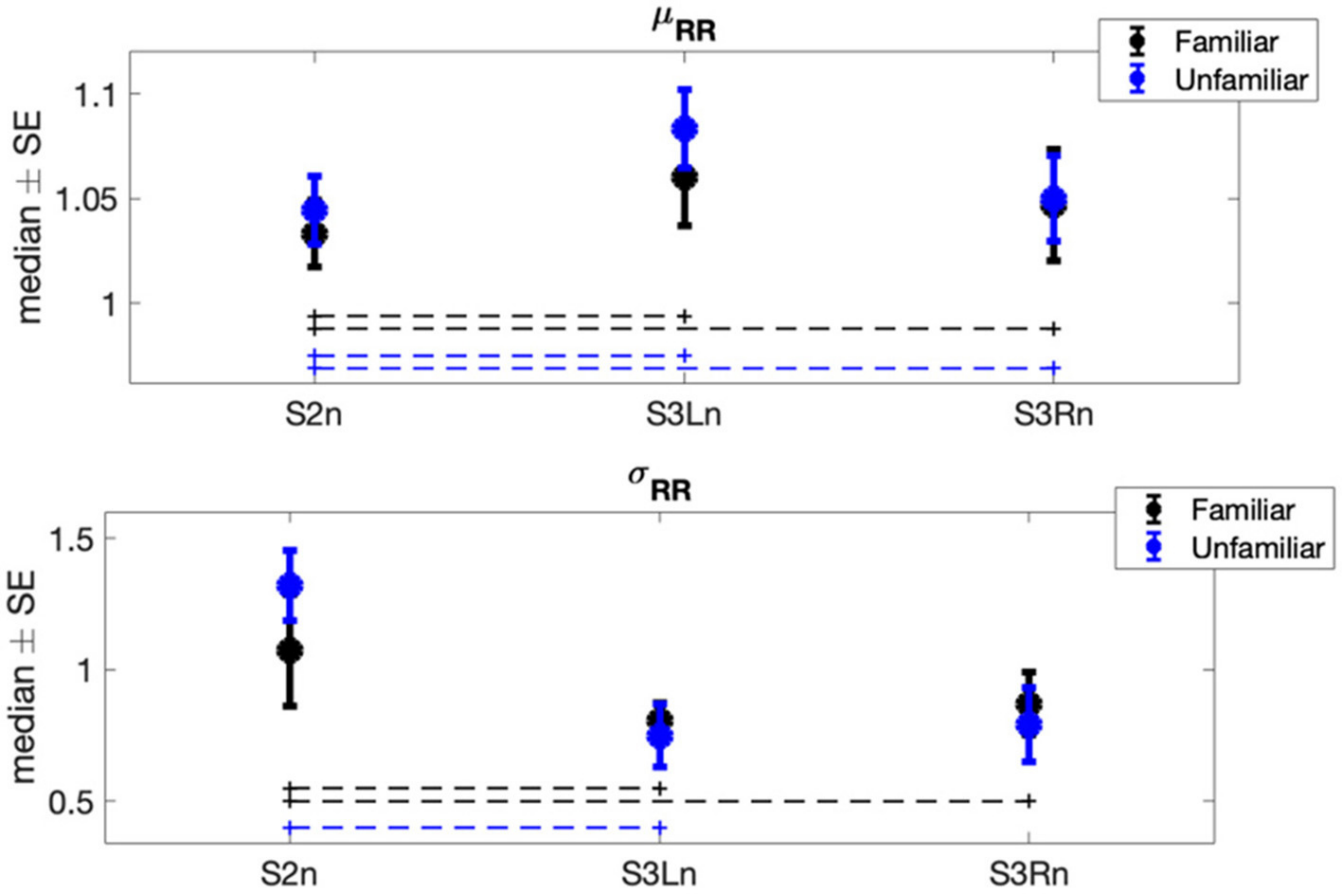
Each error bar represents the median ± standard error (SE) of time-domain normalized features showing at least a significant result in one of the two statistical analyses (i.e., Φ1 or Φ2) in each experimental session. Blue plots are associated with the heart rate variability (HRV) signals recorded during the interaction between horses and the related familiar person; black plots are associated with the HRV signals recorded during the interaction between horses and the related non-familiar person. The dot lines indicate which pair of sessions was significantly different within each group.

**Figure 3:**
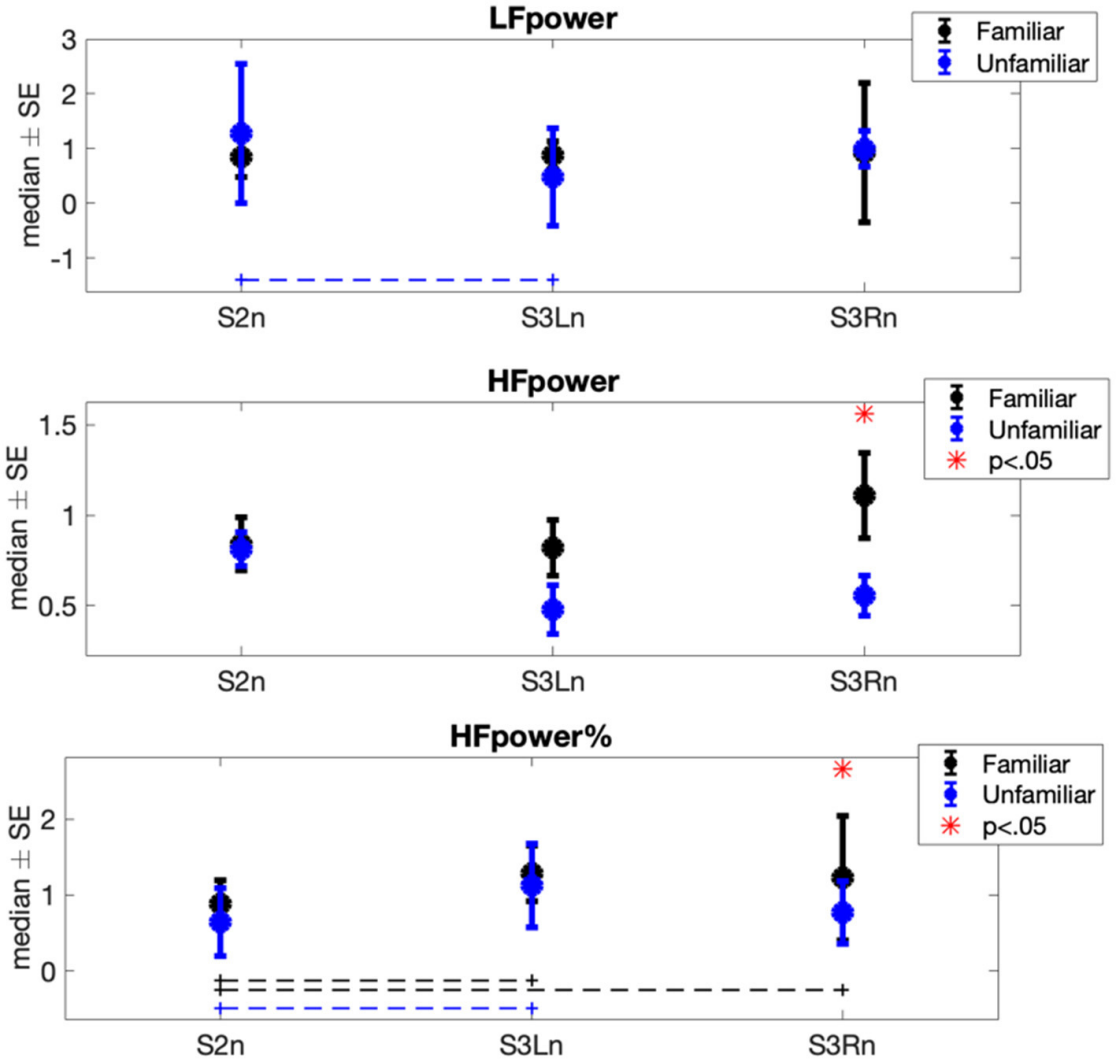
Each error bar represents the median ± standard error (SE) of frequency-domain normalized features showing at least a significant result in one of the two statistical analyses (i.e., Φ1 or Φ2) in each experimental session. Blue plots are associated with the heart rate variability (HRV) signals recorded during the interaction between horses and related familiar person; black plots are associated with the HRV signals recorded during the interaction between horses and the related non-familiar person. The dot lines indicate which pair of sessions was significantly different within each group. The red star shows during which session the statistical comparison between familiar and unfamiliar groups was significant.

The results of Φ1 comparisons (i.e., differences between experimental sessions) showed a significant increase in the horses' mean heart rate (μRR) when both the familiar person and unfamiliar person brushed them (Familiar: p_S2n−S3Ln_ = 0.014, *r* = −0.448, p_S2n−S3Rn_ = 0.002, *r* = −0.475; Unfamiliar: p_S2n−S3Ln_ = 0.008, *r* = −0.457, p_S2n−S3Rn_ = 0.048, *r* = −0.341) (Figure 2). Contrarily, the horses' heart rate standard deviation (σRR) was subjected to a significant decrease in both familiar and unfamiliar interactions (Familiar: p_S2n−S3Ln_ = 0.032, *r* = 0.359, p_S2n−S3Rn_ = 6.66·10^−4^, *r* = 0.538; Unfamiliar: p_S2n−S3Ln_ = 0.003, *r* = 0.475). Concerning the features in the frequency domain (Figure 3), the unfamiliar group showed a significant decrease in LF when the horse was brushed on its left side (S3Ln) in comparison to the exploratory session (S2n) (*post-hoc*-adjusted p_S2n−S3Ln_ = 0.032, *r* = 0.256). Also, a significant increase in HF% was recorded in the same experimental condition (S3Ln), still considering the unfamiliar humans' group (*post-hoc*-adjusted p_S2n−S3Ln_ = 0.021, *r* = −0.354). Moreover, the HF% revealed a significant increase during the grooming phase on both sides of the horses when the familiar set was considered (*post-hoc*-adjusted p_S2n−S3Ln_ = 0.048 *r* = −0.413; p_S2n−S3Rn_ = 0.008, *r* = −0.336). Interestingly, HF% and the HF were the only features that showed noteworthy differences in the Φ2 statistical analysis comparing the two groups, familiar vs. unfamiliar (HF: p_familiar−Unfamiliar_ = 0.003, *r* = 0.430; HF%: p_familiar−Unfamiliar_ = 0.044, *r* = 0.269). In particular, both the median variation of the HF power spectra and percentage power spectra significantly increased when a familiar human was grooming the horse on its right side [i.e., during S3Rn (Table 2, Figure 3)].

## Discussion

Our results show a difference in the horses' heartbeat dynamics during both conditions (familiar vs. unfamiliar person) and through the interacting sessions (presence of a motionless human vs. physical interaction). These findings reflect distinct emotional responses of the animals as implying not only the handler's familiarity with the horse but also the type of interaction he or she may have with it (i.e., contact or contactless). The latter induces a significant decrease in both the mean heart rate (corresponding to an increase in μRR) and its variability (σRR) when horses experience brushing on both sides (Figure 2). This could reflect a general decrease in the horse's arousal level related to the brushing task, independent of the familiarity with the human performing the thus-mentioned task. Indeed, to indicate physiological stress, the average heart rate is actually suitable (16), as it is linked to emotional arousal during both situations, positive and negative. However, since this reaction in μRR and σRR does not change as a function of the familiarity level, it is reasonable to think that they can reflect only two different arousal levels (44), which our protocol itself triggered. Previous studies have indeed proven that petting reduces signs of fear in horses and lowers heart rates (45).

Moreover, it is worth noting that the fixed order of the sessions in our protocol allowed the horses to physically investigate the person prior to the grooming session. It is likely this may have helped the horses decrease their state of alert, thus resulting in a more relaxed condition during the final task.

The most interesting and relevant results are achieved from the statistical comparison between the familiar and unfamiliar interaction. Specifically, when the familiar humans groomed the horses on their right side, both HF and HF% were significantly higher compared to when unfamiliar handlers were in charge of the grooming procedure. This shift of the sympathovagal balance toward a vagal predominance indicates that the horses experienced relaxation when with humans they knew and while interacting with them. Such results can be the overwhelming evidence of the capacity of horses to recognize familiar humans. In fact, these results constitute the objective measure Proops and McComb proposed (1) regarding the capacity of horses to individually recognize familiar people by cross-modally matching multiple information criteria. Moreover, Proops et al. (46) found that horses, after a single encounter with an individual displaying an emotional facial expression, reacted accordingly to the subsequent interaction with that same person in a neutral context, even after 3–6 h. Lansade et al. (6) showed how horses preferred to touch pictures showing the face of their current or previous keeper instead of a novel unknown face during an experimental trial. Specifically, horses were able to recognize the photograph of a familiar keeper even if they had not seen him or her for 6 months. Besides supporting our results, these studies brought up an additional compelling issue [i.e., the associations between emotions and memory. It has been proven that those events that induce positive or negative emotional state are more easily recalled than those considered emotionally neutral (47)]. Our study reveals that the familiarity with the handler is paramount for the horse to feel comfortable, and this is even truer when the interaction involves a physical contact. Therefore, the contact involving familiar humans likely triggered individual-specific emotional memory in tested horses, which, as suggested by physiological dynamics, presumably has a positive valence.

Interestingly, we obtained significant differences between the two familiarity levels only when the handlers physically interacted with the right side of the horse. Indeed, while familiar interactions induced a significant increase in the HF% when contact occurred on both sides of the horse, the grooming performed by the unfamiliar humans showed a significant increase in the HF% only when performed on the left side. In addition, also concerning the HF index, a strong difference between the two familiarity levels is shown only when right-side contact is considered. It is well-known that handling procedures on domestic horses are traditionally practiced on their left side. Therefore, we hypothesized that the approach on the right side constituted an additional stimulus for tested horses, potentially perceived as an unusual handling position and thus contributing to the increase of their discomfort when performed by an unfamiliar handler.

Following the same logic, the behavior of LF appears conceivable. Although LF does not seem to provide an index of cardiac sympathetic activity (48), it is nonetheless affected by the alteration in the sympathovagal balance after the start of the interaction between human and horse. We could, however, speculate that, due to the increase in HF, the decrease in LF may reflect a shift in the sympathetic tone.

It is important to note that our study relies on a strong standardization of experimental protocols. Two main categories of handling tests have been broadly used so far: the presence of a motionless person who remains still in front of the animal and a slow approach toward the horse itself, leading to physical contact (13). A review of literature regarding horses' reactions to stationary or moving humans (49) reveals that physiological signals are frequently linked to this type of handling test, but usually only considering the horses' average heart rate within a short window as a marker. In a few other cases, cardiac activity has been considered an indicator of emotional states of the horses during interactions with familiar and unfamiliar experimenters; however, handling tests in these studies differed from the ones we implement here [i.e., (50, 51)]. The same handling procedures we used were also employed by Fureix et al. (5), analyzing horses' behaviors with unknown or familiar experimenters, but without collecting physiological variables; in the case of Sankey et al. (52), heart rate alone was monitored. Hence, we here combined an interaction task with three different conditions (no interaction, closeness, and physical contact) with a familiar/unfamiliar human handler test, concomitantly evaluating the effects of these situations on horses' HRV. Even though the finest interpretation of animals' emotional reactions benefits from the incorporation of assorted data, such as behavioral and physiological data, we did not consider horses' temperament or reactivity in the present study. Rather, we focused on how long-term relationships with humans may affect horses' emotional state in daily management activities, which generally involve some sort of contact.

The measurement of either the emotional or affective state of an animal is currently of interest in a variety of fields, such as affective neuroscience, evolutionary zoology, comparative psychology, and animal welfare (53). In particular, the investigation of positive emotions and how to prolong positive affective states in animals both represent promising paths for improving animal welfare (21). Broadening the view on interaction with humans, the possibility to comprehend how an animal is experiencing contact with people is invaluable. Animal-Assisted Interventions (AAIs) may be one field that could benefit the most from this kind of approach. The success of AAI itself is in fact strictly dependent on the affiliative nature and on the emotional involvement characterizing the human–animal dyad (54–56). This work may help in selecting the best procedures in terms of the physical approach of the animal involved in the interventions, in accordance with species-specific behavioral features, and it emphasizes the importance of building a relationship, thus not reducing the interaction to the occasional encounters characterizing the therapy.

## Conclusion

Our results suggest that a sequence of positive interactions with the same caretaker represents for horses the probable trigger for experiencing presumed positive emotions during the interaction itself. The novelty of this study lies in the possibility to obtain horses' affective assessments, carried out through the objective analysis of their HRV. The opportunity to effectively measure the emotional state of an animal, in multiple conditions including during contacts with other individuals, paves the way for a broad variety of future studies that set the human perspective to the side so as to prioritize that of the animal.

## Data Availability Statement

The raw data supporting the conclusions of this article will be made available by the authors, without undue reservation.

## Ethics Statement

The studies involving human participants were reviewed and approved by Ethics Committee of the Experimental Zooprophylactic Institute of Venice (IZSVe). The patients/participants provided their written informed consent to participate in this study. The animal study was reviewed and approved by Ethics Committee of the Experimental Zooprophylactic Institute of Venice (IZSVe) CE IZSVe 07/2020. Written informed consent was obtained from the owners for the participation of their animals in this study. Written informed consent was obtained from the individual(s) for the publication of any potentially identifiable images or data included in this article.

## Author Contributions

PB, AL, CS, and AG designed the experiments and collected the data. AG, AL, and EF analyzed the data. CS, AG, LC, and PB wrote the first draft. All authors finalized the manuscript.

## Conflict of Interest

AG, AL, and ES are founding partners of the company Feel-Ing S.r.l. The remaining authors declare that the research was conducted in the absence of any commercial or financial relationships that could be construed as a potential conflict of interest.
